# Construction of engineered corpus cavernosum with primary mesenchymal stem cells *in vitro*

**DOI:** 10.1038/s41598-017-18129-9

**Published:** 2017-12-22

**Authors:** Xiaoshuai Xie, Xiaohang Du, Kailin Li, Yuan Chen, Yong Guan, Xiaofei Zhao, Guangzhu Niu, Yun Luan, Denglu Zhang, Chao Sun, Guanghui Cheng, Jue Wang, Qian Xin, Aibing Xue, Peng Wang, Feng Kong, Xiaoli Liu, Hongwei Wang, Yuqiang Liu, Chuan Tian, Mingzhen Yuan, Shuangde Liu, Shengtian Zhao

**Affiliations:** 1 Department of Urology, The Second Hospital, Shandong University, Shandong, China; 2 Department of Kidney Transplantation, The Second Hospital, Shandong University, Shandong, China; 3 Department of Central Research Laboratory, The Second Hospital, Shandong University, Shandong, China; 4 Shandong University, Affiliated Hospital of Shandong University of Traditional Chinese Medicine, Shandong, China; 5 Key Laboratory for Kidney Regeneration of Shandong Province, Shandong, China

## Abstract

Various methods have been used to reconstruct the penis. The objective of this study was to investigate the feasibility of constructing engineered corpus cavernosum with primary mesenchymal stem cells (MSCs) in a rabbit model *in vitro*. Acellular corporal matrices (ACMs) were obtained from adult rabbit penile tissues through an established decellularization procedure. MSCs were separated, purified, and then seeded on ACMs to construct engineered corpus cavernosum. The seeded ACMs were subsequently cultured in an incubator for 14 days. Histological analyses showed that MSCs seeded on the ACMs had proliferated and were well distributed. Detection of CD31, vWF, smooth muscle actin (SMA), and myosin protein as well as vWF and myosin mRNA revealed that the MSCs had differentiated into endothelial cells and smooth muscle cells. In addition, cell morphology of the engineered corpus cavernosum was directly observed by transmission electron microscopy. This study demonstrated that engineered corpus cavernosum could be successfully constructed using primary MSCs *in vitro*. This technology represents another step towards developing engineered corpus cavernosum *in vitro*.

## Introduction

Conditions such as congenital malformations, trauma, cancer, or other deformities of the penis often require penile reconstruction^1–4^. Many surgical reconstructive procedures, such as using different flap types^5,6^ or implanting cartilages^7^ or prosthetic components^8^, have been attempted to recreate penile function, but the main obstacles^9,10^ to successful reconstruction are surgical complications, including infection, necrosis and shortage of native tissues. Furthermore, corporal function cannot be completely recovered^11,12^. To overcome the limitations associated with penile reconstruction, various tissue engineering^13^ approaches have been considered to potentially restore normal function. Previous researchers have seeded differentiated cells, including smooth muscle cells, endothelial cells^14–16^ and umbilical artery smooth muscle cells^17^, on acellular corporal matrices (ACMs) to reconstitute functional corpus cavernosum. However, owing to the absence of differentiation, single type or combinations of multiple types of seed cells cannot completely restore the function of the corpus cavernosum. The types of cells cultured^18,19^ also increase the complexity of penile reconstruction and applications. In addition, it is difficult to initiate cell culture when the corpora cavernosa are absent. Therefore, stem cells^20^ are considered as a good alternative for reconstructing engineered corpus cavernosum. Nonetheless, autologous embryonic stem cells and other pluripotent stem cells are difficult to acquire. In addition, these cells have the potential to form tumours *in vivo* ^21^, thus restricting their applications. Among adult stem cells, mesenchymal stem cells (MSCs) have been the most widely studied and can be easily acquired by bone marrow aspiration^22,23^ from individual patients. The most important characteristic of these cells is considered to be their capacity to maintain tissue integrity^24^. Combining MSCs with ACMs may allow the large amount of tissues required for corpus cavernosum reconstruction to be generated *in vitro* without inducing morbidity in donor areas. In addition, engineered corpus cavernosum would be biocompatible and eliminate the risk of rejection.

In the present study, we sought to evaluate the use of primary MSCs as seeding cells to construct engineered corpus cavernosum *in vitro*. The seeded ACMs were cultured by a dynamic culturing system to ensure that the seeded cells could obtain sufficient oxygen and nutrients. Finally, we analysed the differentiation of MSCs seeded on ACMs. The findings of this study represent another step towards the construction of engineered corpus cavernosum *in vitro*.

## Results

### Isolation and identification of MSCs

**Figure 1:**
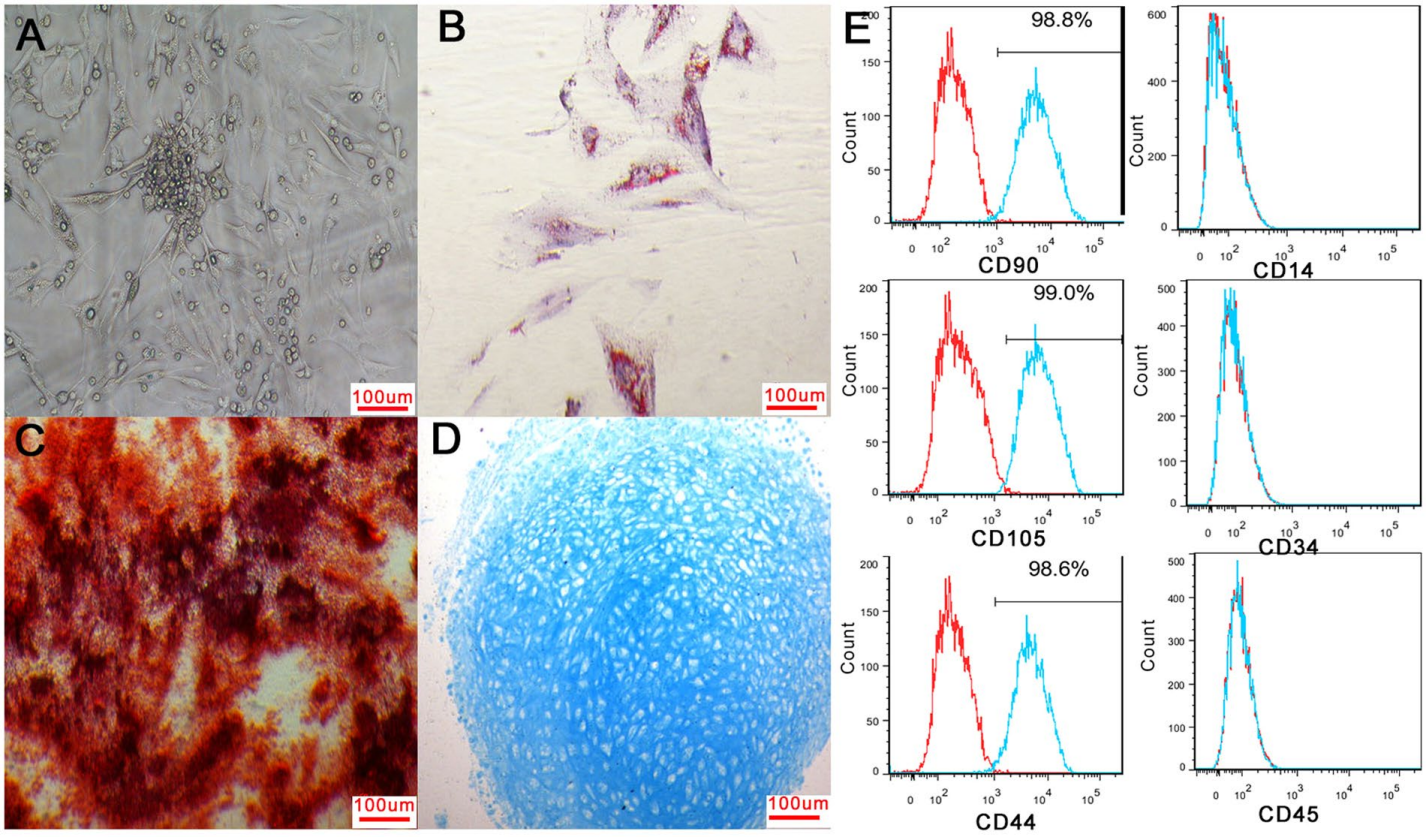
Identification of MSCs. Most primary MSCs were spindle-shaped (**A**). After adipogenic, osteogenetic or chondrogenic induction, cells were positive for oil red O staining (**B**), alkaline phosphatase staining (**C**) or Alcian blue staining (**D**). Flow cytometry showed that cells were negative for CD34, CD14 and CD45 and that the percentages of cells positive for CD44, CD90 and CD105 were greater than 95% (**E**).

MSCs were successfully isolated from bone marrow by the Percoll density gradient centrifugation method, and the MSCs were adherent. Cell colonies formed after seeding for five or six days, and most primary cells were spindle-shaped (Fig. 1 A). Cells reached 80–90% confluency after eight to ten days. The growth curves of the MSCs at passages 1, 3, and 5 were similar and all cells showed a typical “S” shape (Supplementary Fig. S1). After adipogenic induction, oil red O staining revealed red lipid droplets in adipose cells (Fig. 1B). After osteogenetic induction, cells were positive for alkaline phosphatase staining (Fig. 1C). After chondrogenic induction, chondrogenic pellets were coloured blue with Alcian blue staining (Fig. 1D). Phenotype detection by flow cytometry showed that the percentages of cells expressing CD44, CD90 and CD105 were greater than 95% and the percentages of cells expressing CD34, CD14 and CD45 were less than 2% (Fig. 1E). These results suggested a high purity level of MSCs.

### ACM characterization

#### Corpus cavernosum decellularization

**Figure 2:**
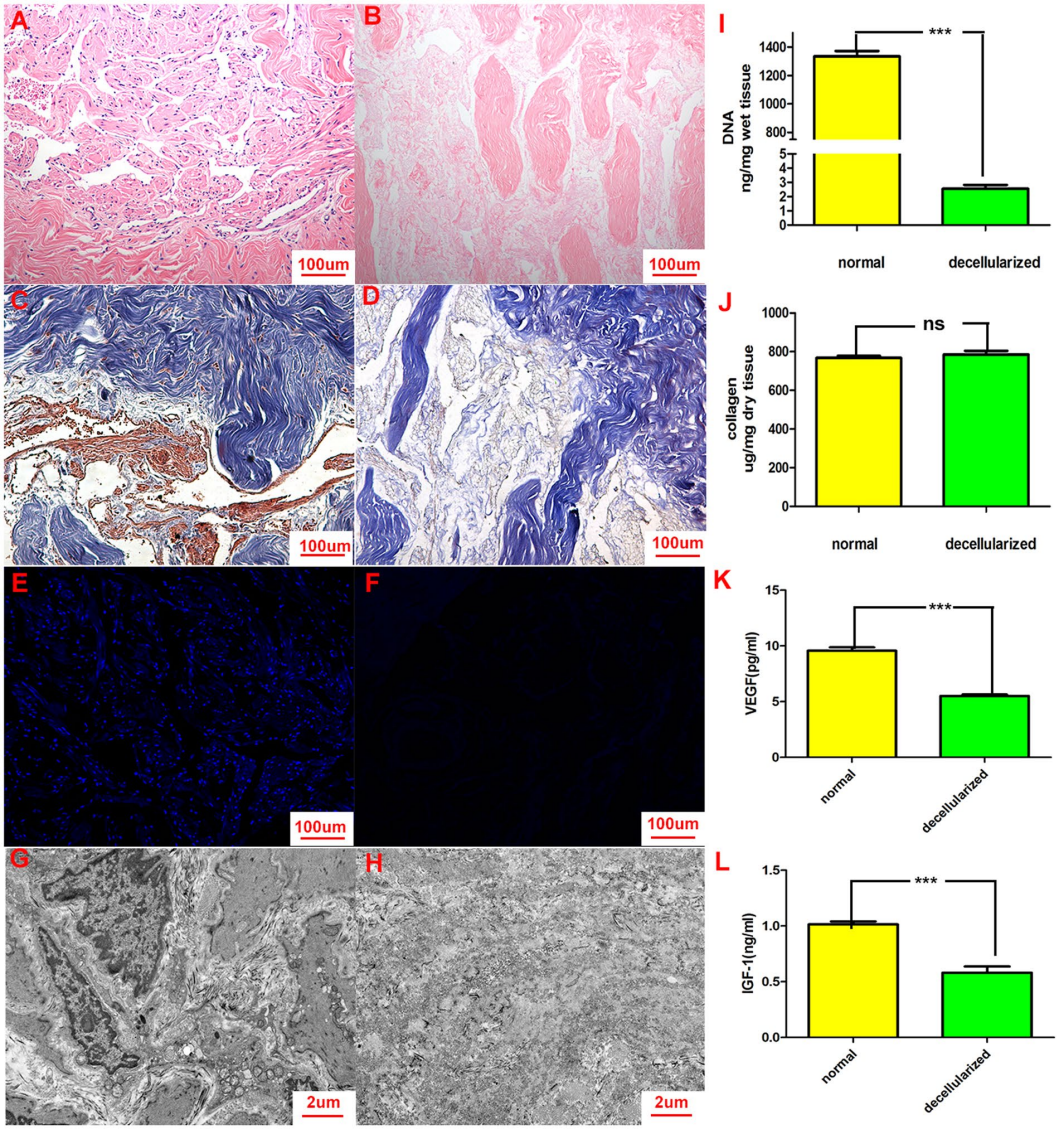
Characterization and histological features of ACMs. Haematoxylin-eosin staining (**A**), Masson trichrome staining (**C**) and DAPI staining (**E**) of normal tissues were shown. Haematoxylin-eosin staining (**B**), Masson trichrome staining (**D**) and DAPI staining (**F**) of ACMs were also performed. The results revealed that no nuclear material was detected and most collagen fibres were preserved. Furthermore, transmission electron microscopy of ACMs (**H**) confirmed that the ACMs contained no residual cellular components, in contrast to normal tissues (**G**). Quantification of DNA in ACMs showed reduced DNA content after decellularization (**I**). Quantification of total collagen was similar to that of normal tissues (**J**). Quantitative assay of cytokines showed that ACMs still contained some cytokines, including VEGF (**K**) and IGF-1 (**L**), but that the concentration of cytokines had decreased.

Using a modified decellularization method, we acquired ACMs that were softer and whiter than native tissues. Compared with haematoxylin-eosin staining (Fig. 2A), Masson trichrome staining (Fig. 2C) and DAPI staining (Fig. 2E) of normal tissues, haematoxylin-eosin staining (Fig. 2B), Masson trichrome staining (Fig. 2D) and DAPI staining (Fig. 2F) of ACMs revealed that no nuclear material was detected. In addition, most collagen fibres were preserved and remained arranged in a loosely packed and congruent order. Furthermore, transmission electron microscopy of ACMs (Fig. 2H) confirmed that the ACMs contained no residual cellular components in contrast to normal tissues (Fig. 2G).

#### DNA and collagen quantification

After 14 days of decellularization followed by washing with PBS for 24 hours, DNA quantification showed that little DNA remained in ACMs compared with normal tissues, thus confirming that the native cellular materials had been removed (Fig. 2I). Collagen quantification revealed that collagen concentration per milligram of ACMs did not significantly differ from that in normal tissues (Fig. 2J).

#### Enzyme-linked immunosorbent assay (ELISA) for cytokines

ACMs still contained some cytokines, including VEGF (Fig. 2K) and IGF-1 (Fig. 2L), but the concentration of these cytokines was reduced compared with normal tissues.

### Histological and cell proliferation analyses

**Figure 3:**
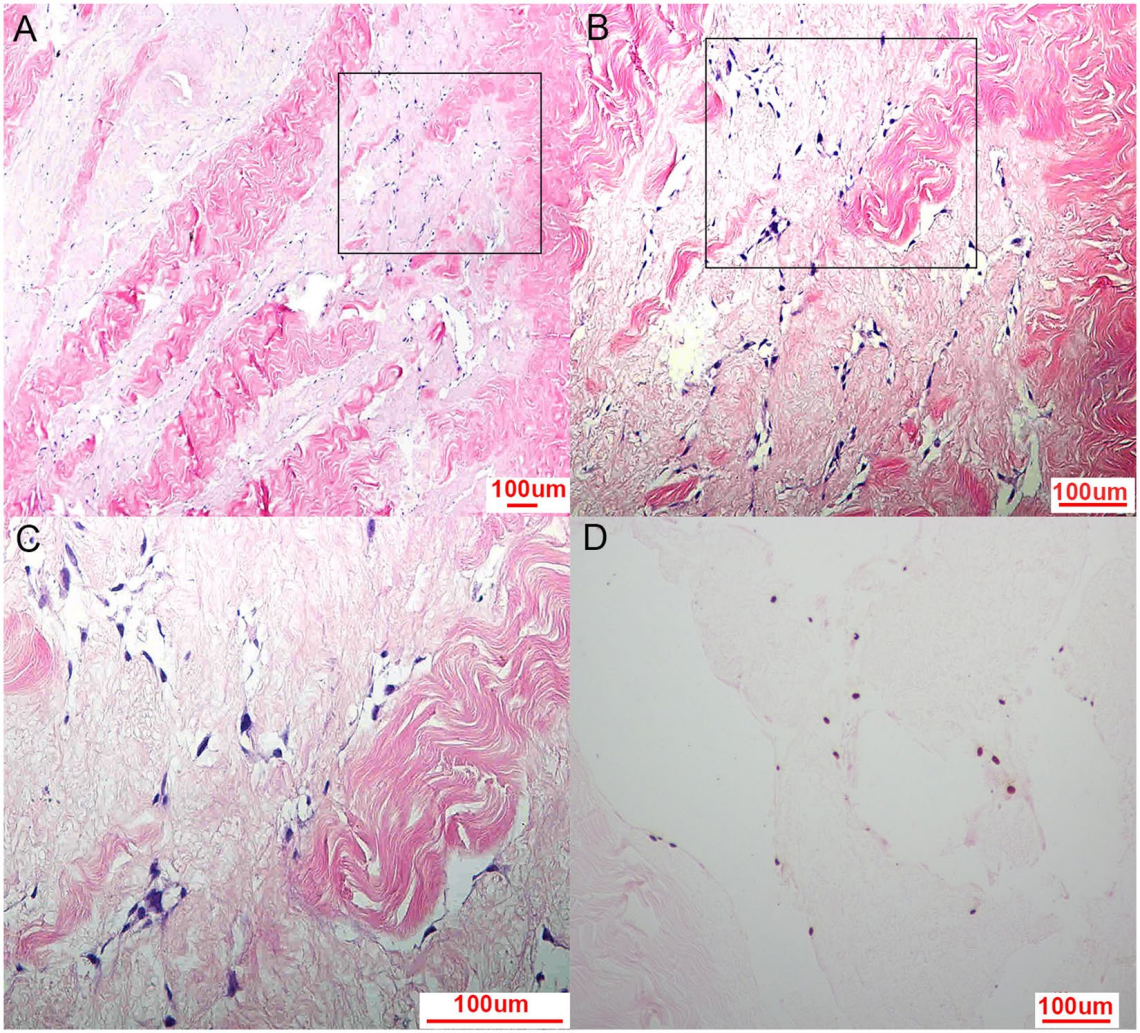
Histology of engineered corpus cavernosum and cell proliferation analysis. Haematoxylin-eosin staining of engineered corpus cavernosum showed that numerous cells distributed and grew well after 14 days of culture *in vitro* (**A,B,C**). Nuclei of ACMs that had proliferated were coloured brown by EdU uptake (**D**), indicating cell proliferation in ACMs.

MSCs were seeded on the ACMs and cultured for 2 weeks *in vitro*. The seeded ACMs were examined by haematoxylin-eosin staining after 2 weeks. The results showed that numerous MSCs had distributed and grown well in the engineered corpus cavernosum (Fig. 3A–C). MSCs seeded in the ACMs were detected by EdU absorption from the culture medium. The nuclei of MSCs that had proliferated were coloured brown (Fig. 3D), indicating cell proliferation in the ACMs.

### Immunofluorescence and transmission electron microscopy analyses

**Figure 4:**
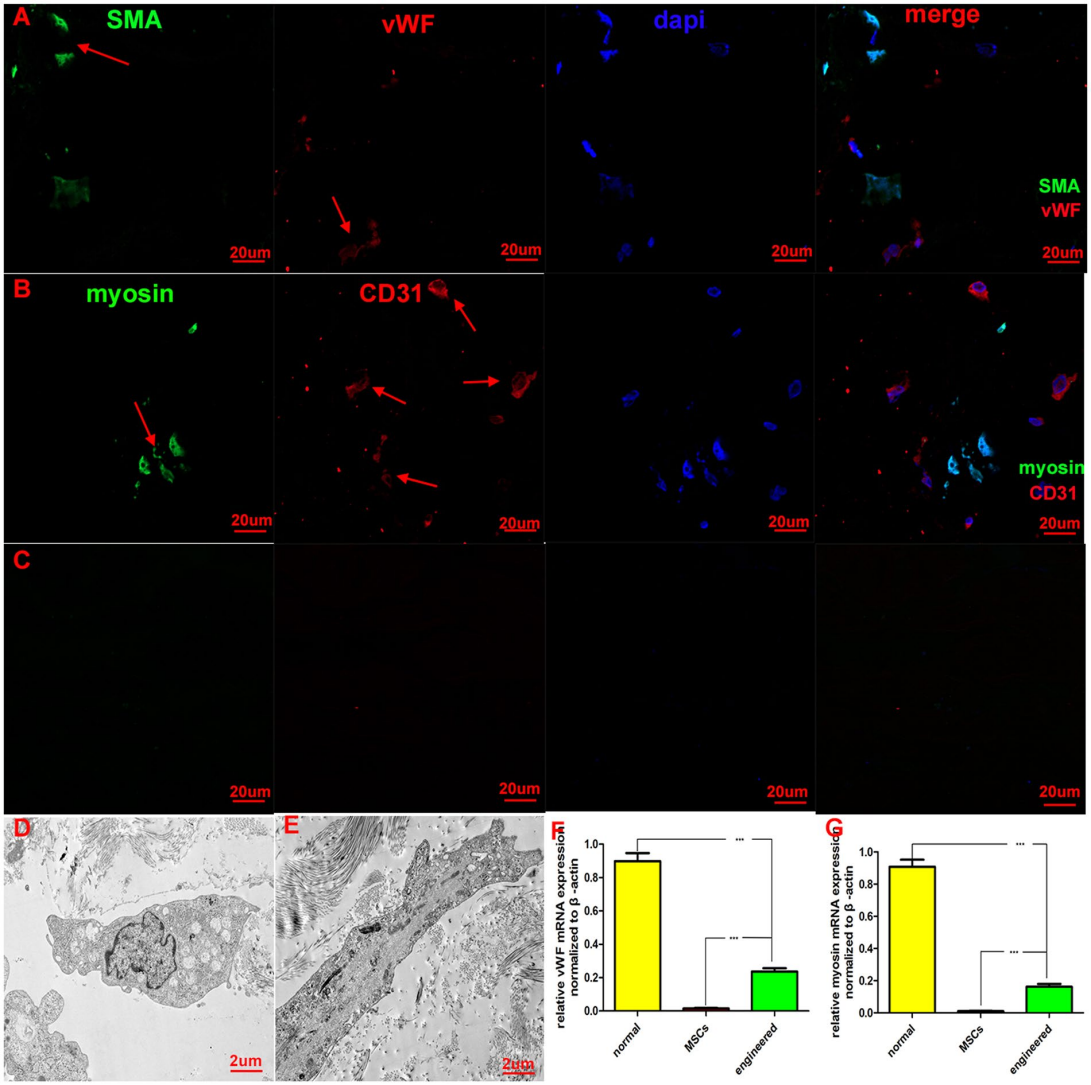
Differentiation of MSCs detected by immunofluorescence, reverse transcriptase and quantitative polymerase chain reaction and transmission electron microscopy. Immunofluorescence for vWF (**A**; red) and CD31 (**B**; red) was positive, indicating the existence of endothelial cells. Smooth muscle cells were detected by immunofluorescence of SMA (A; green) and myosin (**B**; green). Immunofluorescence of ACMs (**C**) as a control group was also showed. Morphology of endothelial cells (**D**) and smooth muscle cells (**E**) was detected in the engineered corpus cavernosum by transmission electron microscopy, which indicated that MSCs seeded on ACMs had differentiated into endothelial cells and smooth muscle cells. The relative mRNA expression levels of vWF (**F**) and myosin (**G**) were high in normal tissues and very low in MSCs. In addition, there were significant differences in mRNA expression levels between the engineered corpus cavernosum and MSCs.

Immunofluorescence revealed that some seeded cells stained positively for vWF (Fig. 4A; red) and CD31 (Fig. 4B; red) and that some cells stained positively for smooth muscle actin (SMA) (Fig. 4A; green) and myosin (Fig. 4B; green). Immunofluorescence of unseeded ACMs (Fig. 4C) was shown as a control. Transmission electron microscopy of the engineered corpus cavernosum confirmed the existence of endothelial cells (Fig. 4D) and smooth muscle cells (Fig. 4E). Transmission electron microscopy and immunofluorescence studies revealed the presence of endothelial cells and smooth muscle cells in the engineered corpus cavernosum.

### mRNA expression

Reverse transcriptase and quantitative polymerase chain reaction was performed to detect the mRNA expression of vWF and myosin. The results showed that vWF (Fig. 4F) and myosin (Fig. 4G) mRNA expressed in normal tissues and in the engineered corpus cavernosum but not in MSCs. Although vWF and myosin mRNA were detected in the engineered corpus cavernosum, the expression was much lower than that in normal tissues. These data suggest that some MSCs had differentiated into smooth muscle cells and endothelial cells.

### Protein expression

**Figure 5:**
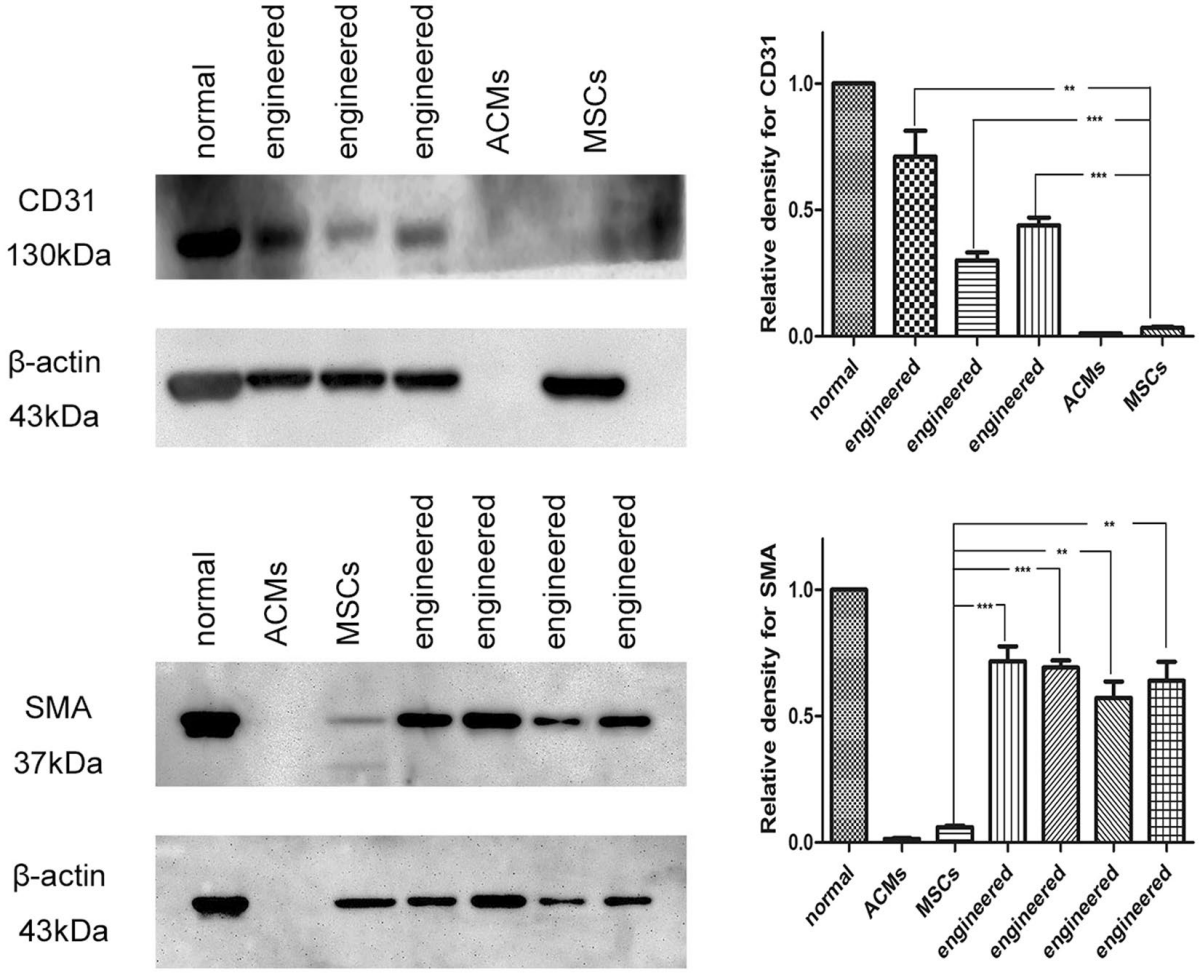
Protein expression of CD31 and SMA in different groups. CD31 and SMA were minimally expressed in MSCs and ACMs but more highly expressed in engineered corpus cavernosum and normal groups. The relative densities of CD31 and SMA normalized to native tissue revealed that the engineered tissues markedly differed from the MSCs. The results showed that a portion of the MSCs had differentiated into endothelial cells and smooth muscle cells.

Western blot was applied to detect the protein expression of CD31 and SMA. The result revealed negative CD31 expression in MSCs and ACMs and positive CD31 expression in normal tissues and the engineered corpus cavernosum. SMA expression was negative in ACMs and weakly positive in MSCs but strongly positive in normal tissues and the engineered corpus cavernosum (Fig. 5). Primary data for western blot was also showed (Supplementary Fig. S2). The results showed that CD31 and SMA were deficient in MSCs but expressed in normal tissues and the engineered corpus cavernosum, thus confirming the existence of endothelial cells and smooth muscle cells. The relative density of CD31 and SMA normalized to native tissue revealed that the engineered tissues were quite different from MSCs.

## Discussion

Using ACMs to construct anatomical and functional corpus cavernoum has been proposed as a potential treatment for penile diseases^13^. In recent years, various regenerative approaches^14–17^, including seeding cells on biodegradable polymer scaffolds or decellularized matrices, have been attempted to develop functional engineered corporal tissue. Adult cells such as smooth muscle cells, epithelial cells and endothelial cells have the ability to proliferate quickly *in vitro* but can’t differentiate. Besides, these cells lack corresponding cytokines which are important in the the construction of engineered corpus cavernosum. Embryonic stem cells and other pluripotent stem cells are difficult to acquire and have the potential to form tumours *in vivo* ^20,21^, which restricts their applications. The application of MSCs as seeding cells in regenerative medicine has made penile reconstruction possible^25^. MSCs have the capacity to differentiate into smooth muscle cells and endothelial cells which are the main components of the corpus cavernosum^26,27^. In addition, the engineered corpus cavernosum using autologous MSCs can eliminate the risk of rejection and has considerable potential for large-scale production.

ACMs were obtained from adult rabbit penile tissues through an established decellularization procedure^15^. No complete nuclear material remained while most collagen fibres were preserved in the ACMs after treating with Triton X-100^15–17^, as confirmed by the histological results (Fig. 2). We further quantified the DNA and collagen content in the ACMs. These results revealed that we preserved most collagen fibres and fulfilled the criteria of less than 50 ng/mg residual DNA (Fig. 2I,J). Therefore, we successfully acquired ACMs that were appropriate for the construction of engineered corpus cavernosum.

ACMs have been proven to provide an excellent environment for cell survival, attachment, proliferation and differentiation for constructing engineered corpus cavernosum^28,29^. To assess the potential of ACMs for seeding cells, we performed ELISA to quantify the levels of VEGF and IGF-1. The results (Fig. 2K,L) showed that the ACMs retained a portion of the cytokines found in native tissues. These cytokines are important for cells, thus providing support for the reconstruction of engineered corpus cavernosum using ACMs.

In previous studies, the ACMs were seeded with cells and re-transplanted to the rabbits^15–17^. Then, the function of the engineered corpus cavernosum was detected after several weeks *in vivo*. In the present study, we sought to thoroughly construct the engineered corpus cavernosum with primary MSCs *in vitro*. To achieve this goal, the seeded ACMs were cultured for 14 days by using a shaker spinning at 40 RPM in an incubator to ensure that the MSCs seeded on the ACMs acquired sufficient oxygen and nutrients. The engineered corpus cavernosum examined by haematoxylin-eosin staining after 2 weeks showed that numerous cells had distributed and grown well (Fig. 3A–C). In addition, DNA synthesis of the seeded cells was directly measured by detecting proliferation (Fig. 3D), revealing that the cells had proliferated in the ACMs.

Smooth muscle cells and endothelial cells are the main cells of the corpus cavernosum and play an important role in maintaining and regulating penile function. A certain quantity of smooth muscle cells and endothelial cells is necessary for the reconstruction of engineered corpus cavernosum. Adult cells such as smooth muscle cells, endothelial cells and umbilical artery smooth muscle cells have been combined with ACMs to develop engineered corpus cavernosum^14–17^. However, owing to the absence of differentiation, single type or combinations of multiple types of seed cells cannot completely recover the function of corpus cavernosum. MSCs have been applied in the construction of engineered corpus cavernosum for their ability to differentiate into smooth muscle cells and endothelial cells. Although engineered corpus cavernosum using MSCs can lead to partial recovery of normal corporal function according to intracorporal pressures^23^, the effects of ACMs on the differentiation of MSCs have not been elucidated. To clarify the effects of ACMs on the differentiation of MSCs, the seeded ACMs were cultured *in vitro* for 14 days. Reverse transcriptase and quantitative polymerase chain reaction, Western blot and immunofluorescence were performed to detect the differentiation of MSCs seeded in ACMs. Immunofluorescence revealed positive vWF, CD31, SMA and myosin expression (Fig. 4A,B), indicating the existence of endothelial cells and smooth muscle cells. mRNA expression of vWF and myosin showed that vWF (Fig. 4F) and myosin (Fig. 4G) mRNA expressed in normal tissues and in the engineered corpus cavernosum but not in MSCs. Protein expression analyses (Fig. 5) revealed CD31 and SMA expression in engineered corpus cavernosum and normal groups but little expression in MSC and ACM groups. Significant RNA and protein levels indicative of smooth muscle cells and endothelial cells were detected in engineered corpus cavernosum. Furthermore, morphology of endothelial cells (Fig. 4D) and smooth muscle cells (Fig. 4E) in engineered corpus cavernosum was directly observed by transmission electron microscopy. Thus, we concluded that the ACMs provided an excellent microenvironment for cell survival, attachment, and differentiation and that the MSCs seeded on the ACMs had differentiated into smooth muscle cells and endothelial cells. Therefore, engineered corpus cavernosum was successfully constructed with primary MSCs *in vitro*.

Our study also had some limitations. First, the primary MSCs we used were obtained from bone marrow of 4-month-old male rabbits. It is known that there is variability in MSC differentiation potential across donors. Factors such as origin, donor age and microenvironment which might affect the differentiation potential of MSCs^30–32^ were not considered in our experiment. In addition, gender differences of stem cells on therapeutic efficiency had aroused much attention in recent years^33–36^. The differentiation of these cells will be unpredictable when MSCs from female rabbits are seeded on the ACMs. If the engineered corpus cavernosum could be constructed with primary MSCs from female rabbits, this technique will provide convenience for female patients undergoing transsexual operations in clinic. Second, the quantity of smooth muscle cells and endothelial cells in normal tissues was not able to be achieved in the engineered corpus cavernosum. One reason may be that the concentration of cytokines was reduced compared with native tissue. Another reason is that only a part of MSCs have differientiated into smooth muscle cells and endothelial cells. The other MSCs may have differentiated to fibroblasts or still remain to be MSCs. In order to reach the quantity of cells in normal tissues, exogenous cytokines could be added in the future to promote cell adhesion and differentiation. The type and quantity of cytokines which are added to maintain a proper proportion of endothelial cells and smooth muscle cells need further study. Third, the ACMs were 4-mm-thick layers, which cannot be used for entire penile transplantation. The seeding methodology and culture system must be improved to accommodate larger tissue samples. The dynamic nature of the culture method should be further adapted from simply stirring to using more sophisticated pumps to mimic penile erection in order to train the smooth muscle cells and tighten the endothelial layer. Fourth, we investigated the differentiation of MSCs, while the function of the engineered corpus cavernosum needs to be further considered in organ bath studies. The engineered corpus cavernosum should be re-transplanted into rabbits to test its functions by cavernosometry, cavernosography and mating. Furthermore, throughout the process of MSC differentiation, the signalling pathways affected by the ACMs remain unclear. Additional studies need to be performed.

## Conclusion

Engineered corpus cavernosum was successfully constructed with primary MSCs *in vitro*. MSCs seeded on ACMs differentiated into smooth muscle cells and endothelial cells. While further studies are required, these results are encouraging. This technology represents another step towards developing engineered corpus cavernosum *in vitro*.

## Materials and Methods

### Study design

Experiments were carried out using 60 New Zealand White male rabbits aged 4 months with weights ranging from 2.5–3 kg. The ACMs were obtained from 30 rabbit penile tissues. 20 groups of primary MSCs were harvested and expanded from additional 20 rabbits. The penile tissues of the remaining 10 rabbits were used as normal controls. The ACMs of 10 rabbits were used to characterize the ACMs. The ACMs of the other 20 rabbits were seeded with MSCs to construct the engineered corpus cavernosum *in vitro*. Each group of MSCs was only seeded on the responding group of ACMs. All rabbits were grouped randomly. Protein and mRNA expression of the engineered corpus cavernosum was analysed after 2 weeks. To ensure reliability and accuracy, the ACMs or the engineered corpus cavernosum used to detect protein and mRNA expression were from different donor rabbits.

### Isolation and culture of primary MSCs

The animal experimental protocol was approved by the Animal Ethics Committee of the Second Hospital of Shandong University. All surgical procedures were performed according to the Guide for the Care and Use of Laboratory Animals. The MSCs were prepared according to a modified method of the Percoll density gradient centrifugation method. Briefly, bone marrow was obtained from rabbit tibial plateau and added to the surface of the Percoll separating medium. White cloudy mononuclear cells on the separating medium were collected after centrifugation. The cells were then cultured in rabbit mesenchymal stem cell medium in a 37 °C incubator until reaching 80% confluence. After digestion, centrifugation, and resuspension, the cells were expanded until sufficient cell numbers were available for detecting or seeding on the acellular matrices. The percentages of cells positive for CD44, CD90, CD105, CD34, CD45 and CD14^22,37^ were analysed in third-generation MSCs using flow cytometry. Cells at passages 1, 3, and 5 were cultured at the same number in 3.5-cm-diameter petri dishes. Three dishes of cells at different passages were then randomly selected to count the numbers of cells, and the average from the second day to the eighth day was calculated. The data were analysed by GraphPad Prism software, and the growth curve was drawn.

### Osteogenetic induction, adipose induction and chondrogenic induction

Third-generation MSCs were seeded on 6-well plates at a concentration of 3 × 10^5^/mL. When the cells reached 80% confluence, they were treated with osteogenetic induction medium (DMEM/F12, 10% foetal bovine serum [FBS], 0.1 μmol/L dexamethasone, 200 μmol/L vitamin C, 10 mmol/L β-phosphoglycerol) for 21 days for differentiation into osteoblasts or adipogenic induction medium (DMEM/F12, 10% FBS, 1 μmol/L dexamethasone, 200 μmol/L antifani, 0.5 mmol/L IBMX, 10 μg/mL insulin) for 14 days for differentiation into adipocytes. The third-generation MSCs were also adjusted to a concentration of 5 × 10^5^/mL at this time. The cell suspension was added to 15-mL polypropylene culture tubes in aliquots of 0.5 mL and centrifuged. The supernatant was not aspirated and the pellet was not resuspended. Finally, the cells were treated with chondrogenic induction medium (L-DMEM/F12, 10% FBS, 10 μg/L TGF-β3, 0.1 μmol/L dexamethasone, 50 μmol/L vitamin C, 6.25 mg/L insulin) for 21 days for differentiation into chondrocytes. The differentiated cells were identified by alkaline phosphatase staining, oil red O staining or Alcian blue staining respectively.

### ACM preparation

ACMs were prepared by an established method^15^, successfully fulfilled the criteria for removal of native cellular materials (DNA content at or below 50 ng DNA/mg tissue)^38^. Briefly, male rabbits were killed via an air embolism. Corpora cavernosa were dissected through longitudinal incisions in the tunica albugineas and cleared of extra soft tissues. They were then cut into 4-mm-thick slices. The corporal tissues were washed with distilled water in a closed flask, placed on a shaker in a super clean bench for 24 hours to allow partial cell lysis and then treated with 1% Triton X-100 and 0.1% ammonium hydroxide under continuous shaking for 14 days to remove the remaining cells. Finally, the matrices were washed with PBS for 24 hours and then processed with DMEM/F12 medium for another 24 hours. Samples of tissue fragments were stained with haematoxylin-eosin and DAPI to ensure that no remaining cellular components were detected.

### ACM characterization

#### Basic histological analysis

Cell clearance but preserved collagen fibrils were expected in the ACMs. ACMs were fixed in 4% paraformaldehyde, dehydrated, embedded in paraffin, and cut into 4-µm-thick cross-sections. Haematoxylin-eosin staining, Masson staining and DAPI staining were performed to estimate clearance of cells and the preservation of collagen fibrils. In addition, ACMs were also observed by transmission electron microscopy.

#### DNA quantitation

A TIANamp genomic DNA kit was used to quantify DNA content in ACMs and normal tissues according to the manufacturer’s instructions. Briefly, the samples were broken into pieces for cell suspension and centrifugation, and the depositions were then incubated at 56 °C until completed dissolved by proteinase K. An assay was then performed in convenient spin-column format according to the manufacturer’s instructions. Finally, the extracts were characterized spectrophotometrically (NanoDrop 1000; Thermo Scientific). Absorbances at 260 nm (RNA) and 280 nm (DNA) were measured to estimate the yield and purity of nucleic acids, and the results were expressed as nanograms of DNA per milligram of tissue.

#### Collagen quantitation

A Sircol soluble collagen assay was used to quantify soluble collagen according to the manufacturer’s instructions. Briefly, tissues were lyophilized. At a pepsin concentration of 0.1 mg/mL of 0.5 M acetic acid and 4 °C, the enzyme activity was effective to release the tissue collagen into solution. Sircol dye reagent was then added to the test samples. They were mixed and placed in a gentle mechanical shaker for 30 minutes and then centrifuged at 12,000 RPM for 10 minutes. The supernatant was discarded, and the remains were washed with ice-cold acid-salt wash reagent. Finally, alkali reagent was added to release and recover the collagen-bound dye. A microplate reader was used to measure the absorbance at 550 nm. All concentrations were determined on the basis of a standard curve generated in parallel, and values were normalized to original tissue dry weight. The results were expressed as microgram of collagen per milligram of dry tissue.

#### Enzyme-linked immunosorbent assay for VEGF and IGF-1

VEGF and IGF-1 in ACMs and normal tissues were assayed using the VEGF and IGF-1 ELISA kits (Cusabio). Tissues were homogenized in 1 mL PBS and stored overnight at -20 °C. After two freeze-thaw cycles, the homogenates were centrifuged. The supernatant was removed and assayed immediately according to the manufacturer’s instructions. Briefly, samples were added to each well and incubated for 2 hours at 37 °C. After removing the liquid of each well, biotin antibody (1X) was then added to each well, and the samples were incubated for 1 hour at 37 °C. Horseradish peroxidase avidin (1X) was added to each well after washing, and the samples were incubated for another 1 hour at 37 °C. TMB substrate was then added to each well followed by incubation for 20 minutes at 37 °C before adding the stop solution. The concentrations of cytokines including VEGF and IGF-1 were assayed with a microplate reader at 450 nm.

### Cell seeding

The ACMs were transferred to 96-well plates, and each ACM was placed in an individual well. MSCs were trypsinized, centrifuged, resuspended and injected into the ACMs at multiple sites using a 22-gauge needle at a concentration of 30 × 10^6^/mL to ensure that a sufficient number of cells were injected into the ACMs to construct the engineered corpus cavernosum. The seeded ACMs were then preserved in a 37 °C incubator for 6 hours to allow the cells to aggregate and attach to the ACMs. Next, the seeded ACMs were transferred to 12-well plates containing 3 mL DMEM/F12 supplemented with 10% FBS and 1% penicillin-streptomycin, which were changed daily. The 12-well plates were placed on a shaker spinning at 40 RPM for 14 days in a 37 °C incubator. The seeded ACM samples were examined by haematoxylin-eosin staining using paraffin-embedded sections every 2 days to observe the state of the seeded cells.

### Cell proliferation in ACMs

A Click-iT^TM^ EdU Colorimetric IHC Detection Kit (Thermo Fisher) was used to monitor the proliferation of cells in the engineered corpus cavernosum. Two days after cell seeding, EdU (1:1000) was added to the culture medium. The engineered corpus cavernosum was removed 12 hours later and fixed with formalin. Cell proliferation was assessed on the paraffin-embedded sections according to the manufacturer’s instructions. Briefly, the slides were immersed into a solution of 3% H_2_O_2_ in PBS for 10 minutes at room temperature to quench endogenous peroxidase enzymes after deparaffinization. The slides were then digested with trypsin-EDTA to aid in antigen retrieval for 15 minutes. Each tissue section was supplemented with 0.5 mL of the Click-iT^TM^ EdU reaction cocktail and incubated for 30 minutes at room temperature. Then, 2 drops of streptavidin-peroxidase conjugate were added to each section followed by incubation at room temperature for 30 minutes in a humidified chamber. Finally, each tissue section was supplemented with 200 μL DAB reaction mixture, rinsed briefly with deionized water and incubated at room temperature for 5 minutes. The results were observed by optical microscopy.

### Immunofluorescence and transmission electron microscope analyses

Immunofluorescence was performed on frozen sections of engineered corpus cavernosum using antibodies against CD31 (1:1000; Abcam) and vWF (1:200; Santa) to test for endothelial cells. Antibodies against SMA (1:200; Abcam) and myosin (1:1000; Abcam) were also used to test for smooth muscle cells. Briefly, engineered corpus cavernosum were cut into 4-µm-thick cross-sections and rinsed well with PBS. They were permeabilized with 1% Triton X-100 in PBS for 10 minutes at room temperature. Then, non-specific antibody binding sites were blocked by incubating in blocking buffer (1% serum) for 1 hour. The sections were incubated with primary antibodies at 4 °C overnight. The sections were then washed and transferred back to the humidified chamber for secondary antibody (Alexa Fluor 488 donkey anti-mouse IgG [H + L], Alexa Fluor 633 donkey anti-goat IgG [H + L]) incubation for 1 hour at room temperature. Last, they were washed and stained with DAPI. Immunofluorescence was observed by confocal microscopy. Immunofluorescence of unseeded ACMs was used as a control. In addition, the state of the cells was observed by transmission electron microscopy.

### Reverse transcriptase and quantitative polymerase chain reaction

Total cellular RNA was extracted from normal tissues, MSCs and engineered corpus cavernosum using TRIZOL according to the manufacturer’s instructions. cDNA was synthesized by using a FastQuant RT Kit (Tiangen). Primer sequences for amplification of vWF and myosin are shown in Table 1. The SYBR Green I assay was used to perform real-time quantitative polymerase chain reaction products. Finally, we used the 2^−ΔΔ^Ct method to analyse the relative mRNA expression levels with β-actin as an internal control.

**Table 1:** Primers for RT-PCR.

gene	primer sequence
vWF	F: 5′-GGAAGAGTGTGATGATTGATGTG-3′ R: 5′-TTCTCCCAGATGTACTCTCC-3′
Myosin	F: 5′-CATCTCTTCCAAGTATGCGG-3′ R: 5′-GTCTTCATCTCCTCCATCTG-3′
β-actin	F: 5′-CGAGATCGTGCGGGACAT-3′ R: 5′-CAGGAAGGAGGGCTGGAAC-3′

#### Western blot

Western blot was applied to detect the protein expression of CD31 and SMA. Tissue lysates were prepared by RIPA for protein expression assay. Protein samples of normal tissues, ACMs, MSCs and engineered corpus cavernosum were resolved by SDS-PAGE and transferred to immobilon polyvinyl difluoride (PVDF) membranes. The blots were blocked with 4% bovine serum albumin (BSA) for 1 h at room temperature and then probed with the primary antibodies against CD31 (1:1000, Abcam) and SMA (1:200, Abcam) overnight at 4 °C. The blots were subsequently incubated with the secondary goat anti-mouse antibodies conjugated with horseradish peroxidase for 1 h at room temperature. The blots were visualized by an ECL system. The relative density of immunoblotting panels normalized to native tissue was analysed by Quantity One software.

#### Statistical analyses

For all assays, at least three independent experiments were performed. The results were expressed as the mean value ± standard deviation (SD) by GraphPad Prism software. The statistical significance of differences between two groups was analysed with two-sided unpaired Student’s t-tests, and the data were deemed to be statistically significant when the P value was less than 0.05.

## Electronic supplementary material

Supplementary information
