# Supplementary material for: Construction of engineered corpus cavernosum with primary mesenchymal stem cells in vitro
Source: Sci Rep. 2017 Dec 22;7:18053. doi: 10.1038/s41598-017-18129-9 (PMC5741727; doi:10.1038/s41598-017-18129-9)

Supplementary information

Title: *Construction of engineered corpus cavernosum with primary mesenchymal stem cells in vitro*

Authors: Xiaoshuai Xie[1], Xiaohang Du[1], Kailin Li[3], Yuan Chen[3], Yong Guan[1], Xiaofei Zhao[3], Guangzhu Niu[1], Yun Luan[3], Denglu Zhang[3], Chao Sun[3], Guanghui Cheng[3], Jue Wang[3], Qian Xin[3], Aibing Xue[1], Peng Wang[5], Feng Kong[3], Xiaoli Liu[2], Hongwei Wang[2], Yuqiang Liu[1], Chuan Tian[2], Mingzhen Yuan[1*], Shuangde Liu[2*], Shengtian Zhao[4,5*]

Authors affiliations:

1.Department of Urology, The Second Hospital, Shandong University, China

2.Department of Kidney Transplantation, The Second Hospital, Shandong University, China

3.Department of Central Research Laboratory, The Second Hospital, Shandong University, China

4.Shandong University, Affiliated Hospital of Shandong University of Traditional Chinese Medicine, China

5. Key Laboratory for Kidney Regeneration of Shandong Province

*Corresponding authors:

Mingzhen Yuan, e-mail: yuanmingzhen2005@126.com

Shuangde Liu, e-mail: liushuangdechina@126.com

Shengtian Zhao, e-mail: zhaoshengtian@sdu.edu.cn

Supplementary information includes:

1. Supplementary figure legends
2. Supplementary figures

Supplementary Fig. S1 legends

Growth curves. The growth curves of MSCs at passage 1, 3, 5 were similar. All of them showed typical "S" shape.

Supplementary Fig. S2 legends

Primary data for western blot. PVDF memberanes were cut into two parts. Proteins of interest and β-actin were visualized by ECL system.

Supplementary Fig. S1

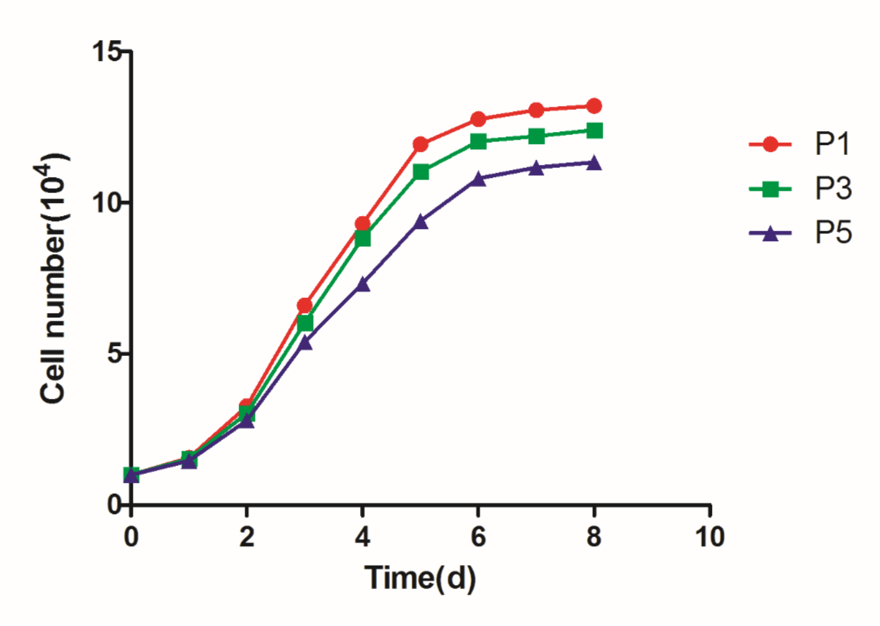

Supplementary Fig. S2

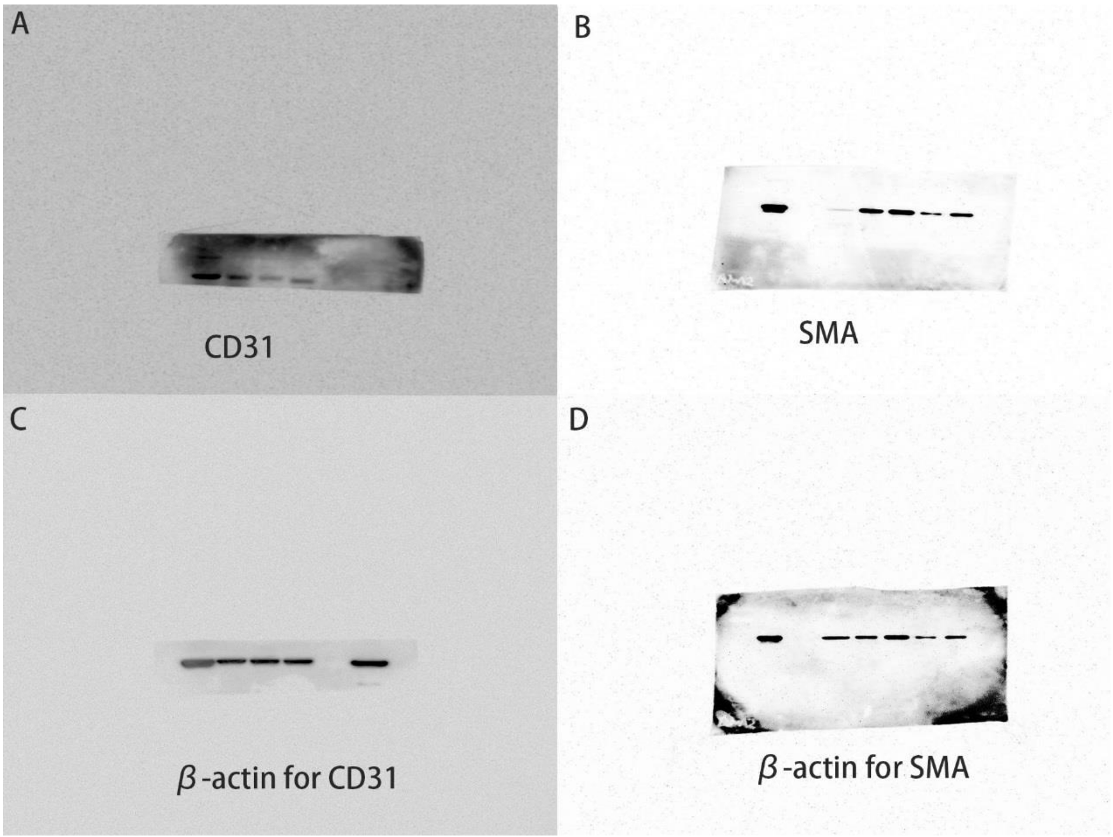

Supplement: Supplementary file 1 — Supplementary information [file 41598_2017_18129_MOESM1_ESM.pdf]
